# Actin Alpha 2 (ACTA2) Downregulation Inhibits Neural Stem Cell Migration through Rho GTPase Activation

**DOI:** 10.1155/2020/4764012

**Published:** 2020-05-16

**Authors:** Ji Zhang, Xuheng Jiang, Chao Zhang, Jun Zhong, Xuanyu Fang, Huanhuan Li, Fangke Xie, Xiaofei Huang, Xiaojun Zhang, Quan Hu, Hongfei Ge, Anyong Yu

**Affiliations:** ^1^Department of Emergency, Hospital of Zunyi Medical University, 563003 Zunyi, Guizhou, China; ^2^Department of Neurosurgery and Key Laboratory of Neurotrauma, Southwest Hospital, Military Medical University (Army Medical University), 400038 Chongqing, China

## Abstract

Although neural stem cells (NSCs) could migrate towards lesions after central nervous system (CNS) injury, the migration ability always is restricted due to the disturbed composition and density of the adhesion ligands and extracellular matrix (ECM) gradient after CNS injury. To date, various methods have been developed to enhance NSC migration and a number of factors, which are affecting NSC migration potential, have been identified. Here, primary NSCs were cultured and the expression of actin alpha 2 (ACTA2) in NSCs was determined using reverse transcription polymerase chain reaction (RT-PCR) and immunostaining. Next, the role of ACTA2 in regulating NSC migration and the potential mechanism was explored. Our results demonstrated that ACTA2 expressed in NSCs. Meanwhile, downregulated ACTA2 using siRNA inhibited NSC migration through hindering actin filament polymerization via increasing RhoA expression and decreasing Rac1 expression. The present study might enrich the basic knowledge of ACTA2 in NSC migration and open an avenue for enhancing NSC migration potential, subsequently providing an intervention target for functional recovery after CNS injury.

## 1. Introduction

Stem cells (SCs) are a subtype of unspecialized cells with the capacity of self-renewal and differentiation into one or more developmental cell linage(s) and have aroused great attention for tissue regeneration [1]. Neural stem cells (NSCs) could be activated, *de novo* proliferated, migrated towards the lesions, directed to three major central nervous system (CNS) cell type: neurons, astrocytes, and oligodendrocytes, and integrated into the injured regions to regulate tissue homeostasis and repair after CNS injury [1–3]. Migration is one of the main characteristics of NSCs. A previous study has indicated that NSCs could proliferate in the subventricular zone (SVZ), one region of the adult brain that persists neurogenesis throughout adult life [4], but only a small number of proliferated NSCs migrate to the lesions after ischemic stroke [5], suggesting that limited functional recovery might be due to insufficient functional NSCs in lesions. Herein, exploring factors influencing NSC mobility is a significant issue using NSCs in cell replacement therapies after CNS injury.

Cell migration relies on actin filament polymerization at the leading edge. Previous studies have demonstrated that actin-associated proteins shootin1, cortactin, cofilin, Arp2/3, ezrin, and slingshot are engaged in actin waves to improve cellular polarity formation and migration [6–8]. NSCs are one of the most common motile cell subtypes. Our previous study has indicated that *α*-actinin 4 (ACTN4) promotes actin filament polymerization and therefore enhances NSC migration [1]. Furthermore, our study also indicates that CDC42 activation facilitates NSC movement through promoting actin filament polymerization [3]. Hence, molecules affecting actin filament polymerization of NSCs might direct NSC motility.

Actin alpha 2 (ACTA2), also known as alpha smooth muscle actin (*α*-SMA), encodes an isoform of globular actin (G-actin), which is assembled into filamentous actin (F-actin) to allow actin cytoskeleton remodeling and to drive cell migration [9, 10]. A previous study has shown that ACTA2 downregulation remarkably impaired human hepatic stellate cell migration via its actin-binding domain [11]. Moreover, researches also indicate that ACTA2 potentiates metastatic potential of human lung cancer cells via enhancing actin filament assembling [12, 13]. In addition, investigations delineate that ACTA2 could improve the migration potential of fibroblasts and mesenchymal stem cells [14–16]. Whether ACTA2 expresses in NSCs and its role in NSC migration still remain unexplored.

In this present study, we examined the role of ACTA2 in NSC migration and explored its underlying mechanism. First, the primary NSCs were cultured and the expression of ACTA2 in NSCs was determined using reverse transcription polymerase chain reaction (RT-PCR) and immunostaining. Then, the role of ACTA2 in regulating actin filament polymerization and the potential mechanism was explored. The aim of this study is to look for factors influencing NSC migration and elucidate the possible underlying mechanism(s), which might enrich the basic theory related to NSCs and provide an intervention target for enhancing NSC migration potential, therefore promoting functional recovery after various neurological diseases and injuries.

## 2. Materials and Methods

### 2.1. Animals

Embryonic C57BL/6 mice were purchased from the laboratory of the Third Military Medical University (Amy Medical University). All animal procedures were performed in accordance with China's animal welfare legislation for protection of animals used for scientific purpose and were approved by the local authorities of the Third Military Medical University for the laboratory use of animals.

### 2.2. Primary NSC and Brain Microvascular Endothelial Cell (BMEC) Culture

A total number of 25 embryonic day 14.5 C57BL/6 mice were employed to obtain primary NSCs as previously described [2, 17]. Briefly, the cerebral cortices were washed twice with Dulbecco's Modified Eagle's Medium (DMEM, Hyclone, Logan, Utah). Then, samples were washed with 10% fetal bovine serum (FBS, vol/vol, Hyclone, Logan, Utah) twice to inhibit the activity of trypsin after incubation in 0.25% trypsin-EDTA (Hyclone, Logan, Utah) at 37°C for 30 min. Then, the tissues were triturated by a fire-polished Pasteur pipette and passed through a 70 *μ*m Nylon cell strainer (BD Falcon, San Jose, CA) after they were washed twice with DMEM. Cell suspensions were cultured in enrichment medium-DMEM/F12 medium supplemented with 2% B27 (Gibco, Grand Island, NY), 20 ng/ml recombinant murine epidermal growth factor (EGF, PeproTech, Rocky Hill, NJ), and 20 ng/ml recombinant murine fibroblast growth factor-basic (FGF, PeproTech, Rocky Hill, NJ) at 37°C under 5% CO_2_ humidified condition. For NSC passage, neurospheres were collected by centrifugation at the speed of 300 rpm, dissociated in StemPro Accutase Cell Dissociation Reagent (Gibco, Grand Island, NY), and grown in enrichment medium as described above. Y27632 was purchased from Sigma-Aldrich (St. Louis, MO, USA), and the working concentration was 30 *μ*M as previously reported [18].

For differentiation, NSCs were firstly seeded on 10 *μ*g/ml poly-L-ornithine- (PO-) precoated coverslips and then incubated in differentiation medium-DMEM/F12 medium supplemented with B27 (Gibco, Grand Island, NY) and 1% glutamax (Gibco, Grand Island, NY) for 10 days as previously reported [19]. The passage of NSCs used for all experiments in the present study was from passage 3 to 5.

BMECs were purchased from OBiO Technology Co., Ltd. (Shanghai, China) and cultivated in the medium recommended by the supplier.

### 2.3. Immunofluorescence

Neurospheres or cells adhered to PO-precoated coverslips were incubated in 4% paraformaldehyde for 10 min at room temperature and then washed with phosphate buffer saline (PBS, pH ~7.4) for three times. The samples were permeated with 0.5% Triton X-100 PBS for 30 min and blocked by 5% bovine serum albumin (BSA) for 2 h after washing three times. Thereafter, the samples were incubated in primary antibodies, goat anti-Nestin (1 : 100, Santa Cruz Biotechnology, CA, USA), rabbit anti-MAP-2 (1 : 100, Proteintech Group, Inc., Beijing, China), rabbit anti-glial fibrillary acidic protein (GFAP, 1 : 100, Abcam, Cambridge, UK), mouse anti-Olig2 (1 : 100, Milipore Corp., Billerica, MA, USA), rabbit anti-*α*-Smooth Muscle Actin (ACTA2) (1 : 100, Beyotime, Beijing, China), and mouse anti-Tubulin (1 : 100, Beyotime, Beijing, China) for 10–14 h at 4°C. After washing, relative fluorescence secondary antibodies were incubated at room temperature for 2 hours. Cell nuclei were counterstained with 4′-6-diamidino-2-phenylindole (DAPI, Sigma-Aldrich, St. Louis, MO) for 10 min at room temperature. Then, coverslips were mounted onto glass slides and the images were captured by a confocal microscope (Carl Zeiss, LSM780, Weimar, Germany) and examined using Zen 2011 software (Carl Zeiss, Weimar, Germany).

### 2.4. Actin Filament Polymerization Detection

Actin filament polymerization was detected as previously described [2, 3]. Briefly, neurospheres were incubated in 4% paraformaldehyde for 10 min at room temperature and then washed with PBS three times. Thereafter, the samples were incubated in Alexa Fluor 488-conjugated phalloidin reagents (Life Technologies, Waltham, MA, USA) at room temperature for 30 minutes. After mounting onto glass slides, images were visualized with a confocal microscope (Carl Zeiss, LSM780, Weimar, Germany) and measured using Zen 2011 software (Carl Zeiss, Weimar, Germany).

### 2.5. Reverse Transcription Polymerase Chain Reaction (RT-PCR)

Total RNA was extracted from NSCs and brain microvascular endothelial cells (BMECs) using a RIzol reagent (Ambion by Life Technologies, Carlsbad, CA, USA) according to the manufacturer's instructions, and contaminating DNA was eliminated with RNase-free DNase (Qiagen, Valencia, CA). For reverse transcription, 2 *μ*l RNA per sample was reverse transcribed using Premix Taq (Takara Taq Version 2.0 plus dye, Takara Bio Inc., Tokyo, Japan) in a total volume of 20 *μ*l according to the manufacturer's instructions. The primers used were as follows: ACTA2: 5′-GGACGTACAACTGGTATTGTGC-3′ (forward) and 5′-TCGGCAGTAGTCACGAAGGA-3′ (reverse) and GAPDH: 5′-GGC CCC TCT GGA AAG CTG TG-3′ (forward) and 5′-CCA GGC GGC ATG GCA GAT C-3′ (reverse). The annealing temperature for PCR was 60°C and carried out for 28 cycles. PCR products were electrophoresed with 2% agarose gel electrophoresis and visualized by Gold view staining (Solarbio, Solarbio Science & Technology Co., Ltd., Beijing, China). Bands were analyzed using ChemiDoc XRS+ System (Bio-Rad, California, USA).

### 2.6. ACTA2 siRNA Transfection

ACTA2-specific siRNA (sc-43591) was purchased from Santa Cruz Biotechnology (CA, USA). ACTA2-specific siRNA were transfected into NSCs using Lipofectamine™ 3000 Transfection Reagent (Invitrogen, Waltham, MA, USA) according to the manufacturer's instructions. The same amount of scramble siRNA and Lipofectamine™ 3000 transfection reagent was served as the negative control. The transfection efficiency was determined by RT-PCR and western blot.

### 2.7. NSC Migration Assays

NSCs were passaged and digested into single cells, and then, they were cultured in different groups. After culturing for 3 days, neurospheres were seeded on PO-precoated 24-well plates for the propagation of NSC migration out of neurospheres. Images were captured after 12 h by a phase-contrast microscopy. The migration distance of NSCs from the edge of the neurospheres was measured by Image-Pro Plus 6.0 software.

### 2.8. Western Blot

Samples in different groups were homogenized with RIPA (Beyotime, Beijing, China) supplemented with protease and phosphatase inhibitors (Roche, Indianapolis, IN, USA). After centrifugation at 14000g for 30 min at 4°C, the supernatant was collected and concentration was determined using the enhanced BCA Protein Assay Kit (Beyotime, Beijing, China). Equal quality of protein was separated by 10% SDS-PAGE under reducing conditions and electroblotted to polyvinylidene difluoride (PVDF) membranes (Roche, Indianapolia, IN, USA). Then, the membranes were blocked in TBST (0.5% Tween-20 in Tris-buffered saline) containing 5% (*w*/*v*) nonfat dry milk (Boster Biological Technology, Wuhan, China) at room temperature for 2 hours. Afterward, the membranes were incubated in primary antibodies, rabbit anti-*α*-Smooth Muscle Actin (ACTA2, 1 : 1000, Beyotime, Beijing, China), rabbit anti-RhoA (1 : 1000, Cell Signaling Technology, Danvers, MA), rabbit anti-Rac1 (1 : 1000, Cell Signaling Technology, Danvers, MA), mouse anti-GADPH (1 : 1000, Santa Cruz Biotechnology, CA, USA), and mouse anti-active RhoA (Neweast, Bath, UK) overnight at 4°C. After washing, the membranes were in the relative horseradish peroxidase- (HRP-) conjugated secondary antibody (Boster Biological Technology, Wuhan, China) at room temperature for 2 hours. Then, bands were visualized by ChemiDoc™ XRS^+^ imaging system (Bio-Rad, California, USA) by WesternBright ECL Kits (Advansta, Menlo Park, CA, USA). Densitometric measurement of each membrane was determined using Image Lab™ software (Bio-Rad, California, USA). GAPDH was served as an internal control.

### 2.9. Statistical Analysis

All data were expressed as mean ± SEM and statistical analyses were performed using SPSS 19.0 software (SPSS, Inc., Chicago, IL, United States). Multiple comparisons were performed by one-way analysis of variance (ANOVA) and followed by Turkey's post hoc test. A *p* < 0.05 was considered to be statistically significant.

## 3. Results

### 3.1. Primary NSC Isolation and Characteristics

**Figure 1:**
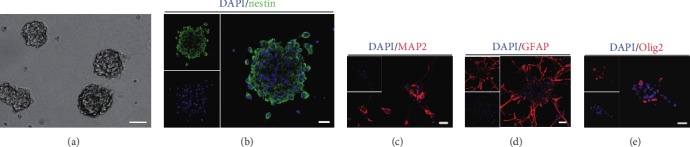
Primary NSC culture and characteristics. (a) Cultured cells exhibited a growth pattern of free floating neurospheres. Scale bars: 100 *μ*m. (b) Immunostaining indicated that most of cultured cells expressed nestin. Scale bars: 20 *μ*m. (c) Immunostaining showed that cultured cells held the potential of differentiation into MAP^+^ cells. Scale bars: 20 *μ*m. (d) Immunostaining demonstrated that cultured cells bore the ability of differentiation into GFAP^+^ cells. Scale bars: 20 *μ*m. (e) Immunostaining delineated cultured cells possessed the capacity of differentiation into Olig2^+^ cells. Cell nuclei were counterstained with DAPI in blue. Scale bars: 20 *μ*m.

For primary NSC culture, neocortical tissues were dissected and harvested from E14.5 C57BL/6 mice. The neurospheres was obviously observed after 3 days cultured in the enrichment culture medium (Figure 1(a)). Meanwhile, most of cells expressed nestin, a marker of NSCs, using immunostaining (Figure 1(b)). To determine the differentiation potential of cultured cells, cells were incubated in a differentiation medium for 7-10 days. The results showed that cells held the capacity of differentiation into neurons (MAP2^+^) (Figure 1(c)), astrocytes (GFAP^+^) (Figure 1(d)), and oligodendrocytes (Olig2^+^) (Figure 1(e)). These results revealed that cultured cells were NSCs and had the ability of proliferation and differentiation into both neuronal and glial (astrocytes and oligodendrocytes) lineages.

### 3.2. ACTA2 Expressed in Primary NSCs and Played an Essential Role in NSC Migration

**Figure 2:**
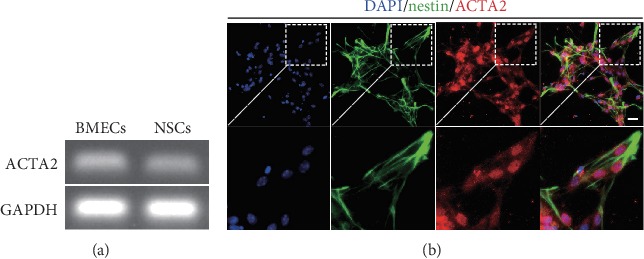
ACTA2 expressed in NSCs. (a) RT-PCR showing that ACTA2 mRNA expressed in NSCs with BMECs as a positive control. (b) The immunostaining images demonstrated the co-labeled of the nestin (red) and ACTA2 (green) in NSCs. Cell nuclei were counterstained with DAPI in blue. Scale bars: 50 *μ*m.

To certify whether ACTA2 expresses in NSCs, we firstly identified ACTA2 mRNA expression using RT-PCR. The results showed that ACTA2 mRNA expressed in NSCs with BMECs as a positive control (Figure 2(a)). Then, the coimmunostaining of ACTA2 and nestin was performed to evaluate ACTA2 protein expression in NSCs and the results delineated that ACTA2 expressed in NSCs.

**Figure 3:**
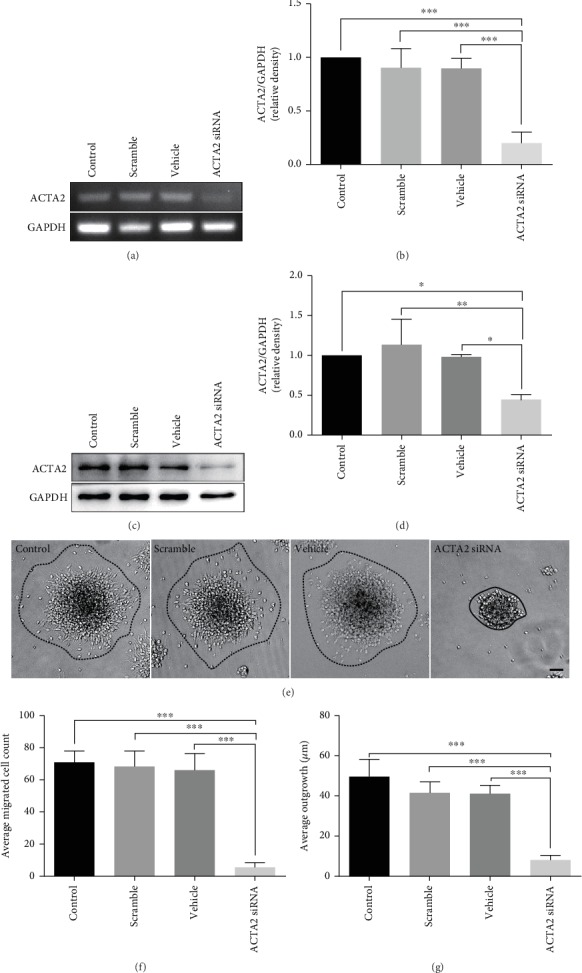
ACTA2 downregulation inhibited NSC migration. (a) RT-PCR showing ACTA2 mRNA expression with ACTA2 siRNA transfection. (b) Quantification of ACTA2 mRNA expression from (a) (*n* = 3 for each group). ^∗∗∗^*p* < 0.001, one-way ANOVA followed by Tukey's post hoc test. (c) Bands showing ACTA2 protein expression with ACTA2 siRNA transfection. (d) Quantification of ACTA2 protein expression from (c) (*n* = 3 for each group). ^∗^*p* < 0.05, ^∗∗^*p* < 0.01, one-way ANOVA followed by Tukey's post hoc test. (e) Representative images of NSC migration from neurospheres plated on PO-precoated 24-well plates under different conditions after 12 hours. Scale bar: 100 *μ*m. (f) Bar graph summarized the number of migration cells from neurospheres in each group (*n* = 6 for each group). ^∗∗∗^*p* < 0.001, one-way ANOVA followed by Tukey's post hoc test. (g) Bar graph summarized the average outgrowth distance migrating from neurospheres in each group (*n* = 6 for each group). ^∗∗∗^*p* < 0.001, one-way ANOVA followed by Tukey's post hoc test.

Next, to explore the role of ACTA2 in NSC migration, ACTA2 siRNA was used to downregulate ACTA2 expression. The results demonstrated that ACTA2 mRNA expression was significantly reduced by ACTA2 siRNA, compared to control, scramble, and vehicle groups (Figure 3(a) and 3(b)). Subsequently, the western blot reconfirmed the results obtained from RT-PCR assays (Figures 3(c) and 3(d)). In addition, the cell number and outgrowth distance emigrating from neurospheres were obviously decreased in the ACTA2 siRNA group, in comparison with control, scramble, and vehicle groups under phase-contrast microscopy (Figures 3(e)–3(g)). Together, these results indicated that ACTA2 downregulation inhibited NSC migration.

### 3.3. Actin Filament Polymerization Was Engaged in ACTA2 Manipulating NSC Migration

**Figure 4:**
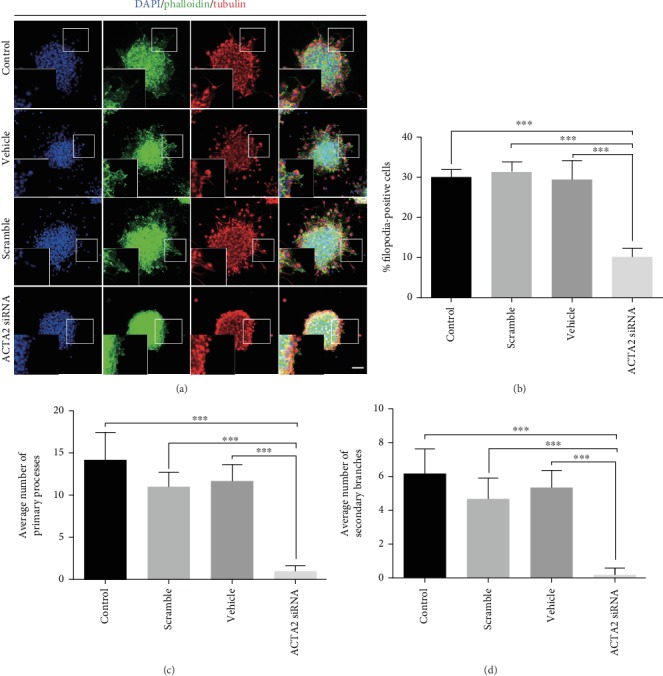
ACTA2 downregulation inhibited filopodia formation. (a) Representative immunostaining of tubulin and phalloidin after neurosphere migration for 12 hours in various groups. Cell nuclei were counterstained with DAPI in blue. Scale bar: 100 *μ*m. (b) Quantification of the percent of filopodia formation in each group (*n* = 6 for each group). ^∗∗∗^*p* < 0.001, one-way ANOVA followed by Tukey's post hoc test. (c) The average number of primary processes was summarized in the statistical graph (*n* = 6 for each group). ^∗∗∗^*p* < 0.001, one-way ANOVA followed by Tukey's post hoc test. (d) Quantification of the average number of secondary branches (*n* = 6 for each group). ^∗∗∗^*p* < 0.001, one-way ANOVA followed by Tukey's post hoc test.

To uncover the possible mechanism underlying NSC migration, we hypothesized that actin filament polymerization might engage in this process due to the pivotal role of actin filament polymerization in cell migration. F-actin assembling was assessed using immunostaining of phalloidin and tubulin, a symbol of cage-like microtubule structure [1], to visualize the morphological structure changes in NSCs. The results showed that the percentage of filopodia formation was significantly reduced with ACTA2 downregulation, compared to control, scramble, and vehicle groups (Figures 4(a) and 4(b)). Meanwhile, the average number of leading processes and secondary branches was also evidently decreased in the ACTA2 siRNA group than that in the control, scramble and vehicle groups (Figures 4(a), 4(c), and 4(d)). These results delineated that actin filament polymerization was engaged in ACTA2 manipulating NSC migration.

### 3.4. The Rho Family of Small GTPases Was a Mediator Regulating Actin Filament Polymerization

**Figure 5:**
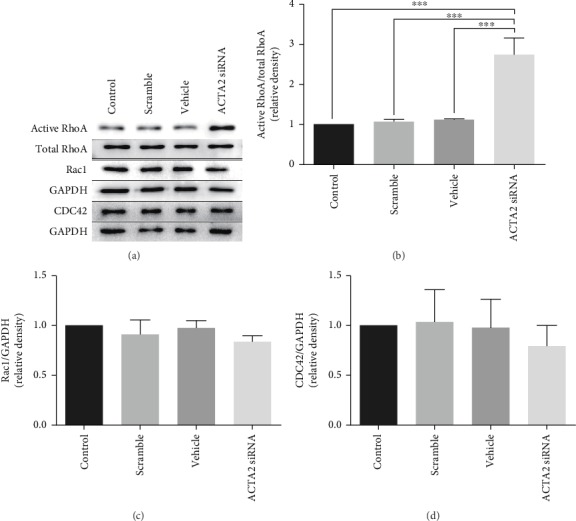
ACTA2 downregulation increased active RhoA and downregulated Rac1 expression. (a) Bands represented the expression of RhoA, Rac1, and CDC42. Total RhoA and GAPDH was used as a loading control. (b) Quantification of active RhoA expression from (a) (*n* = 3 for each group). ^∗∗∗^*p* < 0.001, one-way ANOVA followed by Tukey's post hoc test. (c) Quantification of Rac1 expression from (a) (*n* = 3 for each group). ^∗^*p* < 0.05, one-way ANOVA followed by Tukey's post hoc test. (d) Quantification of CDC42 expression from (a) (*n* = 3 for each group).

The Rho family of small GTPases, including Rho, Rac, and CDC42, is essential for actin filament polymerization in various cell types [20]. To further confirm whether the Rho family of small GTPases is involved in ACTA2 regulating actin filament polymerization to facilitate NSC migration, we assessed the expression of RhoA, Rac1, and CDC42. The results recapitulated that active RhoA expression in the ACTA2 siRNA group was obviously upregulated than that in the control, scramble, and vehicle groups (Figures 5(a) and 5(b)). Rac1 was significantly downregulated in the ACTA2 siRNA group than that in the control, scramble, and vehicle groups (Figures 5(a) and 5(c)). Meanwhile, the CDC42 expression had no significant difference among those groups (Figure 5(a) and 5(d)).

**Figure 6:**
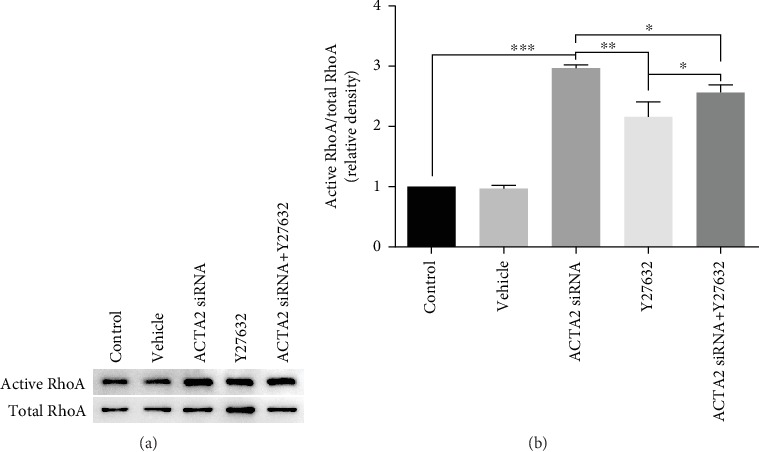
Y27632 partially decreased active RhoA expression induced by ACTA2 downregulation. (a) Bands demonstrated active RhoA expression in various groups. Total RhoA was used as a loading control. (b) Quantification of active RhoA expression from (a) (*n* = 3 for each group). ^∗^*p* < 0.05, ^∗∗^*p* < 0.01, and ^∗∗∗^*p* < 0.001, one-way ANOVA followed by Tukey's post hoc test.

Next, in order to explore the contribution of the Rho GTPase RhoA in ACTA2 mediating NSC migration, NSCs were treated with Y27632, one of the RhoA inhibitors. The western blot results indicated that Y27632 could partially downregulate the expression of active RhoA (Figures 6(a) and 6(b)). Meanwhile, the bands depicted that active RhoA expression was evidently increased when ACTA2-silencing NSCs were treated with Y27632 (Figures 6(a) and 6(b)).

**Figure 7:**
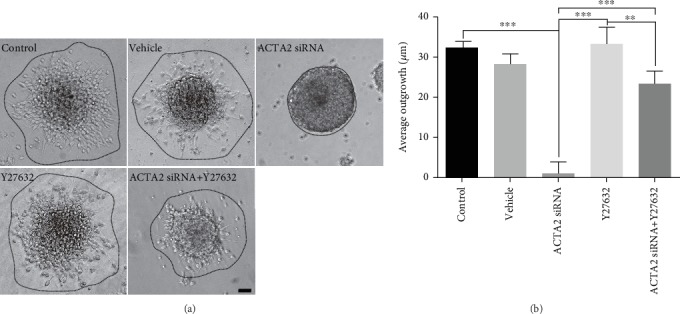
Y27632 partially abrogated inhibitory effect induced by ACTA2 downregulation. (a) Representative images of NSC migration from neurospheres plated on PO-precoated 24-well plates under different conditions after 12 hours. Scale bar: 100 *μ*m. (b) Bar graph summarized the average outgrowth distance migrating from neurospheres in each group (*n* = 6 for each group). ^∗∗^*p* < 0.01, ^∗∗∗^*p* < 0.001, one-way ANOVA followed by Tukey's post hoc test.

Subsequently, we observed the effect of Y27632 on neurosphere migration and the results demonstrated that migration distance was significantly increased in the group with Y27632 treatment than the ACTA2 siRNA group (Figures 7(a) and 7(b)). This enhancement effect was partially abrogated when ACTA2-silencing NSCs were treated with Y27632 (Figures 7(a) and 7(b)).

**Figure 8:**
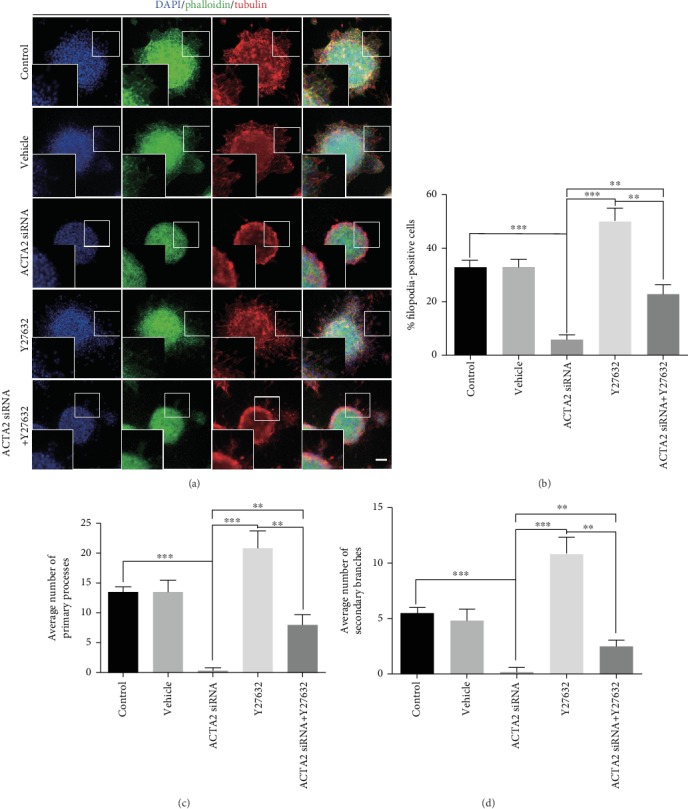
Y27632 partially eliminated inhibitory effect through promoting actin filaments polymerization. (a) Representative immunostaining of tubulin and phalloidin after neurosphere migration for 12 hours in each group. Cell nuclei were counterstained with DAPI in blue. Scale bar: 100 *μ*m. (b) Quantification of the percent of filopodia formation in each group (*n* = 6 for each group). ^∗∗^*p* < 0.01, ^∗∗∗^*p* < 0.001, one-way ANOVA followed by Tukey's post hoc test. (c) The average number of primary processes was summarized in the statistical graph (*n* = 6 for each group). ^∗∗^*p* < 0.01, ^∗∗∗^*p* < 0.001, one-way ANOVA followed by Tukey's post hoc test. (d) Quantification of the average number of secondary branches (*n* = 6 for each group). ^∗∗^*p* < 0.01, ^∗∗∗^*p* < 0.001, one-way ANOVA followed by Tukey's post hoc test.

In addition, to determine the role of Y27632 playing in ACTA2 mediating NSC migration, the immunofluorescence result indicated that the percentage of filopodia formation was partially increased with Y27632 addition in ACTA2 downregulation, compared to the ACTA2 siRNA group (Figures 8(a) and 8(b)). Meanwhile, the average number of leading processes and secondary branches was partially decreased in the ACTA2 siRNA group supplemented with Y27632 than in the ACTA2 siRNA group (Figures 8(a), 8(c), and 8(d)). Together, these results delineated that RhoA was a mediator of manipulating actin filament polymerization resulting from ACTA2 in NSC migration.

## 4. Discussion

In the present study, primary NSCs were cultured and our results demonstrated that ACTA2 expressed in NSCs. Meanwhile, downregulated ACTA2 using siRNA inhibited NSC migration through hindering actin filament polymerization via increasing RhoA expression and decreasing Rac1 expression.

The Rho family of small GTPases, typically including RhoA, Rac1, and CDC42, is a significant family regulating the migration of a bulk of cell types [6, 21]. Here, our results indicated that elevated RhoA resulting from ACTA2 downregulation by ACTA2 siRNA impaired NSC migration, which is line with previous studies [20–23]. Moreover, our results also demonstrated that Rac1, another member of the Rho family of Rho GTPases, was reduced after ACTA2 downregulation using ACTA2 siRNA. Rac1 downregulation is another factor hindering cell migration [24, 25]. Hence, ACTA2 downregulation hinders NSC migration through RhoA upregulation and Rac1 downregulation to dually inhibit actin filament polymerization. We focused our attention on the RhoA signaling pathway as the increased level of RhoA was higher than the decreased level of Rac1. In addition, previous studies have proven that mediation of RhoA regulates proliferation and differentiation of mesenchymal stem cells [26–29], adipose-derived stem cells (ADSCs) [30], muscle stem cells [31], and a bulk of cancer cells [18, 32, 33].

Cytoskeleton rearrangement resulting from RhoA activation holds the ability of affecting NSC characteristics. A previous report has indicated that cytoskeletal rearrangement regulates mesenchymal stem cell (MSC) differentiation into neurogenic subtypes [34]. Moreover, cytoskeleton rearrangement affects adipogenesis due to reorganization of the cells' extracellular matrix (ECM) network microenvironment [35], suggesting that cytoskeletal rearrangement might also affect cell proliferation. Here, our results indicated that ACTA2 mediated actin filament polymerization to regulate NSC migration and cytoskeleton rearrangement. Herein, it is worthy of exploring the role of ACTA2 downregulation causing RhoA activation in NSC proliferation and differentiation in our future research as proliferation and differentiation are two other main features of NSCs.

ACTA2 is a pivotal marker of smooth muscle cells in fibrosis and engaged in vascular contractility and blood pressure homeostasis [36]. A previous study has shown that increased expression of ACTA2 promotes eutopic endometrial stromal cell (euESC) invasion and migration [36]. Meanwhile, research also delineates that inhibition of ACTA2 leads to reduced cellular motility and contraction of myofibroblast during wound healing in vivo, beyond its structural importance in the cell [37]. Furthermore, a previous study certifies that lung cancer cells with high ACTA2 expression exhibit significantly enhanced metastasis, while ACTA2 downregulation remarkably impaired metastasis [12]. To our limited knowledge, it is the first report to find out the expression of ACTA2 in neural cells and its function in NSC migration.

The composition and density of adhesion ligands in the local environment have been shown to be an important variable in controlling cell migration [38], and extracellular matrix (ECM) gradient could direct NSC migration [20]. The composition and density of adhesion ligands, and extracellular matrix (ECM) gradient must be overwhelmingly disturbed after CNS injury, thereafter influencing NSC migration towards lesions to promote local neurovascular repair. Insufficient number of NSCs migrating towards the lesions is a significant factor influencing functional recovery after CNS injury. Various methods have been developed to enhance NSC migration and a number of factors, which are affecting the NSC migration potential, have been identified. Our present research might enrich the basic knowledge of ACTA2 in NSC migration and provide a clue for the use of NSCs with ACTA2 overexpression to promote NSCs in vivo. Meanwhile, the density and gradient still remain elusive. Herein, our next work is to assess the expression level of ACTA2 in the local environment after CNS injury and determine whether the change of local ACTA2 gradient concentration inhibits NSC migration, finally looking for feasible approaches to facilitate NSC migration.

## 5. Conclusions

In sum, the present study demonstrates that ACTA2 is expressed in primary NSCs, and downregulated ACTA2 hinders NSC migration through increasing RhoA expression and decreasing Rac1 expression to inhibit actin filament polymerization, which might enrich the basic knowledge of ACTA2 in NSC migration and open an avenue for enhancing NSC migration potential, subsequently providing an intervention target for functional recovery after CNS injury.

## Data Availability

The data used to support the findings of this study are available from the corresponding author upon reasonable request.
