# Corrigendum to “Actin Alpha 2 (ACTA2) Downregulation Inhibits Neural Stem Cell Migration through Rho GTPase Activation”

**DOI:** 10.1155/2020/5149830

**Published:** 2020-11-05

**Authors:** Ji Zhang, Xuheng Jiang, Chao Zhang, Jun Zhong, Xuanyu Fang, Huanhuan Li, Fangke Xie, Xiaofei Huang, Xiaojun Zhang, Quan Hu, Hongfei Ge, Anyong Yu

**Affiliations:** ^1^Department of Emergency, Affiliated Hospital of Zunyi Medical University, 563003 Zunyi, Guizhou, China; ^2^Department of Neurosurgery and Key Laboratory of Neurotrauma, Southwest Hospital, Third Military Medical University (Army Medical University), 400038 Chongqing, China

In the article titled “Actin Alpha 2 (ACTA2) Downregulation Inhibits Neural Stem Cell Migration through Rho GTPase Activation” [1], the affiliation should be changed from “Department of Emergency, Hospital of Zunyi Medical University, 563003 Zunyi, Guizhou, China” to the correct affiliation “Department of Emergency, Affiliated Hospital of Zunyi Medical University, 563003 Zunyi, Guizhou, China.”

The corrected list of affiliations is shown in the authors' information above.
